# Probing transient memory of cellular states using single-cell lineages

**DOI:** 10.3389/fmicb.2022.1050516

**Published:** 2023-02-07

**Authors:** Abhyudai Singh, Michael Saint-Antoine

**Affiliations:** Departments of Electrical and Computer Engineering, Biomedical Engineering, Mathematical Sciences University of Delaware, Newark, DE, United States

**Keywords:** fluctuation test, cell-state transitions, transient memory, cancer drug resistance, phenotypic heterogeneity, stochastic expression

## Abstract

The inherent stochasticity in the gene product levels can drive single cells within an isoclonal population to different phenotypic states. The dynamic nature of this intercellular variation, where individual cells can transition between different states over time, makes it a particularly hard phenomenon to characterize. We reviewed recent progress in leveraging the classical Luria–Delbrück experiment to infer the transient heritability of the cellular states. Similar to the original experiment, individual cells were first grown into cell colonies, and then, the fraction of cells residing in different states was assayed for each colony. We discuss modeling approaches for capturing dynamic state transitions in a growing cell population and highlight formulas that identify the kinetics of state switching from the extent of colony-to-colony fluctuations. The utility of this method in identifying multi-generational memory of the both expression and phenotypic states is illustrated across diverse biological systems from cancer drug resistance, reactivation of human viruses, and cellular immune responses. In summary, this fluctuation-based methodology provides a powerful approach for elucidating cell-state transitions from a *single* time point measurement, which is particularly relevant in situations where measurements lead to cell death (as in single-cell RNA-seq or drug treatment) or cause an irreversible change in cell physiology.

## 1. Introduction

Advances in single-cell technologies have exposed remarkable differences in phenotype and expression patterns between individual cells within the same isogenic cell population (Raj and van Oudenaarden, 2008; Brandt et al., 2020; Foreman and Wollman, 2020; Lyu et al., 2021; SoRelle et al., 2021; Van Eyndhoven et al., 2021; Topolewski et al., 2022). While some of this variation can be linked to extrinsic factors (i.e., cell-cycle stage, cell size, and local extracellular environment), a growing body of evidence points to the role of stochastic processes inherent to gene expression in driving random fluctuations (noise) in the gene product levels (Süel et al., 2006; Maamar et al., 2007; Eldar and Elowitz, 2010; Singh et al., 2010; Chalancon et al., 2012; Johnston et al., 2012; Neuert et al., 2013; Dar et al., 2014; Magklara and Lomvardas, 2014; Battich et al., 2015; Larsson et al., 2019, 2021; Rodriguez et al., 2019; Ochiai et al., 2020; Fraser et al., 2021). Intercellular phenotypic heterogeneity is physiologically relevant and has important implications for both biology and medicine from driving genetically identical cells to different cell fates (Chang et al., 2008; Losick and Desplan, 2008; St-Pierre and Endy, 2008; Singh and Weinberger, 2009; Thompson et al., 2009; Kim and Sauro, 2012; Norman et al., 2013; Abranches et al., 2014; Balázsi et al., 2014; Torres-Padilla and Chambers, 2014) to facilitating the survival and adaptation of cells to detrimental environmental changes (Kussell and Leibler, 2005; Bishop et al., 2007; Acar et al., 2008; Veening et al., 2008a; Shu et al., 2013; Ackermann, 2015; Doganay et al., 2017; Gasch et al., 2017; Zheng et al., 2018; Evans and Zhang, 2020; Sampaio and Dunlop, 2020; Vasdekis and Singh, 2021).

While single-cell sequencing tools can probe phenotypic heterogeneity within a given cell population, they only provide a static picture of different cell states. Characterizing the dynamics of individual cells transitioning between different states with multi-generational time scales remains a fundamental challenge in advancing the field of single-cell biology. In this regard, a recent innovation leverages the Luria–Delbrück experiment (also called the fluctuation test) in conjunction with mathematical modeling for inferring the switching dynamics between the cellular states. We briefly reviewed this seminal work published 80 years ago.

**Figure 1:**
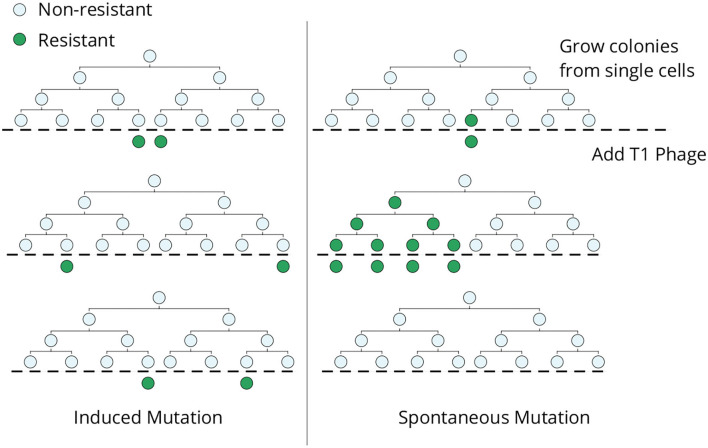
The original Luria–Delbrück fluctuation test. Individual *E. coli* cells were isolated, grown into colonies, and then infected by bacteriophage T1. In the induced mutation hypothesis **(left)**, each cell independently acquires a phage-resistant mutation in response to the infection, and the resulting colony-to-colony variation in the resistant cells would follow a Poisson distribution. In contrast, mutant cells arising spontaneously during lineage expansion prior to viral exposure will lead to large skewed colony-to-colony fluctuations in the number of surviving cells **(right)**—including “jackpot” colonies, where mutations occurred in the early phase of colony expansion leading to a large fraction of resistant cells.

The classical Luria–Delbrück experiment was designed to discriminate whether genetic mutations arise in response to selection or mutations arise randomly in the population in the absence of selection (Figure 1). In an ideal experiment, single *E. coli* cells are grown into clonal colonies and then exposed to the selection pressure (in this case, viral infection by bacteriophage T1). If each bacterial cell has a small and independent probability of gaining a phage-induced mutation, then the number of mutant cells should follow a Poisson distribution across clones (Figure 1, left). Alternatively, if mutations occur randomly, then the number of mutant cells will vary considerably across colonies depending on when the mutation arose in the population expansion (Figure 1, right). The data clearly showed a non-Poissonian skewed distribution for the number of resistant bacteria, validating the hypothesis of pre-existing mutants arising randomly before viral exposure (Luria and Delbrück, 1943). Subsequent mathematical modeling and development of statistical methods allow for the accurate estimation of mutation rates from measured fluctuations across colonies (Koch, 1982; Sarkar, 1991; Jones et al., 1993; Zheng, 1999; Hall et al., 2009; Houchmandzadeh, 2015; Holmes et al., 2017). Apart from its biological significance, this elegant experiment shows how fluctuation-based analysis can reveal hidden random processes that are not directly observable.

While the original Luria–Delbrück experiment considers an *irreversible* change from a non-resistant to resistant phenotype using genetic mutations, this approach can be generalized to consider *reversible* switching between cellular states. We reviewed recent progress in this direction for inferring the transient heritability of cell states (i.e., the number of generations a cell resides in a state before exiting it) from a Luria–Delbrück style experiment. Finally, we highlight several experimental works exploiting this methodology to reveal the plasticity of the drug-tolerant states in cancer cells and discuss its applications to microbiology.

## 2. Fluctuation-test approach to infer cell-state switching

**Figure 2:**
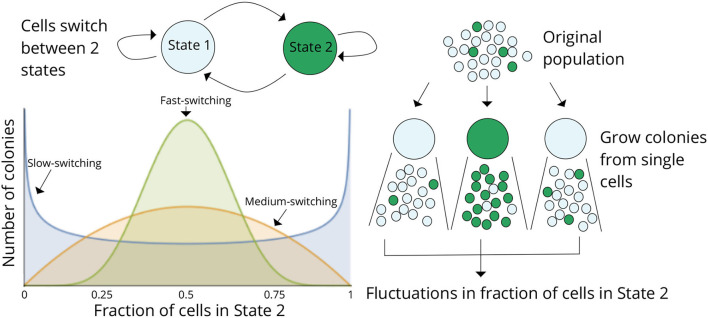
The fluctuation test approach for deciphering switching between two cellular states. Schematic showing cells in two different states (States 1 and 2) together with reversible switching between states and proliferation in each state. Individual cells are randomly chosen from the original population and assayed for the fraction of cells in State 2 after a certain duration of lineage expansion. If switching between states is relatively fast, then the colonies will show similar fractions of State 2 cells as the original population, and variance across colonies will be minimal. On the contrary, if switching is slow, then colony composition will heavily depend on the state of the initial cell, and there would be large colony-to-colony fluctuations based on differences in the initial condition. Thus, statistical fluctuations in colony composition can be exploited to infer the transient heritability of cellular states.

Considering a scenario as in Figure 2 where cells within a population can reside in two states (States 1 and 2), cells proliferate and reversibly switch between states, and the rates of switching determine the transient heritability of a state. Let *f* denote the average fraction of cells in State 2 in the original population. Single cells are randomly drawn from the population (through serial dilutions or FACS sorting or single-cell barcoding) and expanded into colonies. Note that the state of the starting single cell is unobservable, as we only considered a *single* endpoint measurement. After growing the colonies for a certain duration of time, each colony is assayed for the fraction of cells in State 2 (or State 1). The basic idea is that if switching between states is relatively fast (several switches happen in the growth duration), then the fraction of State 2 cells will rapidly equilibrate to *f* in each colony, and colony-to-colony fluctuations will be minimal (Figure 2). In contrast, if switching is slow, then, based on the memory of the initial cell, colonies will primarily be composed of cells in either State 1 or State 2 by generating large colony-to-colony fluctuations (Figure 2). **In essence, fluctuations in colony cell-state composition reveal the timescale of switching, with slower relaxation kinetics driving higher fluctuations**.

In a recent study, we developed several mathematical formulas connecting the magnitude of inter-colony fluctuations to the switching kinetics (Saint-Antoine et al., 2022). We next highlight these formulas that are derived under the following assumptions:

- Cells proliferate at a constant rate *k*_*x*_ that is assumed to be the same irrespective of the cellular state.
- Starting from a single cell, the colony expands exponentially with the average colony size at time *t* being $e^{k_{x}t}$.
- Cells in State 1 transition to State 2 with a rate *k*_1_ and switch back to State 1 with a rate *k*_2_ resulting in the average fraction as follows:

(1) $$\begin{array}{l} f:=\frac{k_{1}}{k_{1}+k_{2}}. \end{array}$$

- The transition rates are assumed to be constants over time and also the same across single-cell colonies.
- The initial cell is chosen randomly from the original bulk population and is either in State 2 with probability *f* or in State 1 with probability 1−*f*.

Let the random process ***f***(*t*) denote the fraction of cells in State 2 at time *t* of colony expansion. Our goal is to quantify statistical fluctuation in ***f***(*t*) as measured by its coefficient of variation:


(2) $$\begin{array}{l} CV_{f}^{2}(t):=\frac{\langle f^{2}(t)\rangle -{\langle f(t)\rangle }^{2}}{{\langle f(t)\rangle }^{2}}, \end{array}$$

Where 〈***f***(*t*)〉 and 〈***f***^2^(*t*)〉−〈***f***(*t*)〉^2^ denote the mean and variance of ***f***(*t*) across colonies, respectively.

### 2.1. Modeling cell proliferation and switching as deterministic processes

In the simplest formulation of this problem, we considered a Bernoulli cell-state assignment of the initial cell where ***f***(0) = 1 with probability *f* (starting cell is in State 2) and ***f***(0) = 0 with probability 1−*f* (starting cell is in State 1). Conditioned on this random initial condition, everything else is modeled deterministically using differential equations. More specifically, the total number of cells over time is $e^{k_{x}t}$, and the fraction of cells in State 2 is given by the following first-order differential equation:


(3) $$\begin{array}{l} \frac{df\left(t\right)}{dt}=k_{1}\left(1-f\right)-k_{2}f. \end{array}$$

Solving Equation (3) results in the solution as follows:


(4) $$\begin{array}{l} f\left(t\right)=f+\left(1-f\right)e^{-\left(k_{1}+k_{2}\right)t}\text{ with probability }f\\ f\left(t\right)=f-fe^{-\left(k_{1}+k_{2}\right)t}\text{ with probability }1-f. \end{array}$$

A straightforward analysis of Equation (4) shows that 〈***f***(*t*)〉 = *f*, which is intuitively expected—the average fraction of cells in State 2 across clones is the same as that in the original population. Note that this time invariant 〈***f***(*t*)〉 is fundamentally different from the classical Luria–Delbrück experiment where the average fraction of mutant cells monotonically increases over time (Luria and Delbrück, 1943).

The colony-to-colony fluctuations can be derived from Equation (4) as follows:


(5) $$\begin{array}{l} CV_{f}^{2}\left(t\right)=\frac{1-f}{f}e^{-\frac{2k_{x}t}{Z}}, \end{array}$$

where the dimensionless quantity


(6) $$\begin{array}{l} Z:=\frac{k_{x}}{k_{1}+k_{2}} \end{array}$$

is a relative measure of the speed of switching with respect to the cell proliferation rate, and *tk*_*x*_ can be interpreted as the average number of generations of colony expansion. Some key features of the fluctuation Equation (5) are as follows:

- At *t* = 0, $CV_{f}^{2}=\left(1-f\right)/f$, which is expected from the Bernoulli distributed cell-state assignment of the initial single cell.
- For a given fixed time point *t* and average fraction *f*, a slower speed of switching will lead to larger values of *Z* and *CV*_*f*_.
- As *t* → ∞, $CV_{f}^{2}\to 0$ with the fraction of State 2 cells converging to *f* across colonies.

From a practical perspective, given a measured value of *CV*_*f*_ at a given time *t* of colony expansion, *a priori* knowledge of the cell proliferation rate and *f* (that can also be estimated from 〈***f***(*t*)〉 = *f*), the switching rates can be estimated by simultaneously solving Equations (1), (5). It is important to point out that this approach for determining transient heritability of cellular states does not require tracking of individual proliferating cells by microscopy over longer period of time to know the exact kinship between cells (Hormoz et al., 2016). To take into account technical fluctuations that are inevitable in an experimental setting, Equation (5) can be modified to


(7) $$\begin{array}{l} CV_{f}^{2}\left(t\right)=\frac{1-f}{f}e^{-\frac{2k_{x}t}{Z}}+CV_{NC}^{2} \end{array}$$

Lu et al. (2021). Here, $CV_{NC}^{2}$ represents technical fluctuations measured using a noise control experiment where random cell populations (of similar size as in the fluctuation test) are drawn from the bulk population and assayed for state fractions. The coefficient of variation of state fractions between these technical repeats determines $CV_{NC}^{2}$. In the limit of rapid switching between cell states (corresponding to non-heritable states), $CV_{f}^{2}\left(t\right)\to CV_{NC}^{2}$.

### 2.2. Modeling cell proliferation and switching as random processes

Perhaps a more accurate modeling approach would be to consider stochasticity in cell proliferation and state switching. Toward that end, for analytical tractability, we considered the cell-cycle time as an independent and identically distributed random variable that follows an exponential distribution with a mean value of 1/*k*_*x*_. While actual cell-cycle times are better captured by Gamma or lognormal distributed random variables, our simulation results show that fluctuations in the fraction of cell states are quite robust to the exact form of the cell-cycle time distribution (Saint-Antoine et al., 2022). The stochastic dynamics of state switching is also modeled as a memoryless process with the time spent in State 2 (State 1) being exponentially distributed with means 1/*k*_2_ (1/*k*_1_), respectively.

Let the integer-value random processes **x**(*t*) and **x**_2_(*t*) denote the total number of cells and the number of cells in State 2, respectively, and now


(8) $$\begin{array}{l} f\left(t\right)=\frac{x_{2}\left(t\right)}{x\left(t\right)}. \end{array}$$

It turns out that, for this stochastic system, one can obtain an exact analytical expression for the statistical moments of **x**(*t*) and **x**_2_(*t*) (see Saint-Antoine et al., 2022) for a detailed derivation of population count moment). To connect fluctuations in absolute cell numbers to fluctuations in ***f***(*t*), two different approximations have been employed.

#### Independent variable approximation

Assuming that the fraction of cells in State 2 in a colony is independent of the colonies, population size


(9) $$\begin{array}{l} \langle x_{2}^{2}\rangle =\langle f^{2}x^{2}\rangle \approx \langle f^{2}\rangle \langle x^{2}\rangle \;\Rightarrow \;\langle f^{2}\rangle \approx \frac{\langle x_{2}^{2}\rangle }{\langle x^{2}\rangle }. \end{array}$$

Exploiting this independence and substituting the moments of absolute cell numbers in Equation (9) results in


(10) $$\begin{array}{l} CV_{f}^{2}\left(t\right)=\left(\frac{2Ze^{tk_{x}\left(\frac{Z-2}{Z}\right)}-2-Z}{\left(2e^{tk_{x}}-1\right)\left(Z-2\right)}\right)\frac{1-f}{f},\;Z:=\frac{k_{x}}{k_{1}+k_{2}}, \end{array}$$

and in the limit *Z* → 2, Equation (10) reduces to


(11) $$\begin{array}{l} CV_{f}^{2}\left(t\right)=\left(\frac{1+2k_{x}t}{2e^{tk_{x}}-1}\right)\frac{1-f}{f}. \end{array}$$

**Figure 3:**
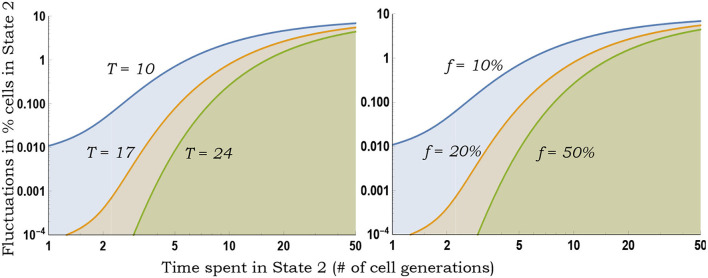
Inferring switching rates from the generalized fluctuation test. **Left**: Colony-to-colony fluctuations in the fraction of State 2 cells as predicted by Equation (10) for fixed *f* = 0.1, as a function of 1/*k*_2_ (average time spent in State 2) for different durations *T* of colony expansion. With decreasing *k*_2_, *k*_1_ is changed to ensure a fixed *f* = 0.1. Slower switching (decreasing *k*_2_) generates larger colony-to-colony fluctuations. **Right**: Same plot as on the left except *T* = 10 (fixed number of cell generations of colony growth) and colony-to-colony fluctuations are plotted for different fractions *f*.

**Figure 4:**
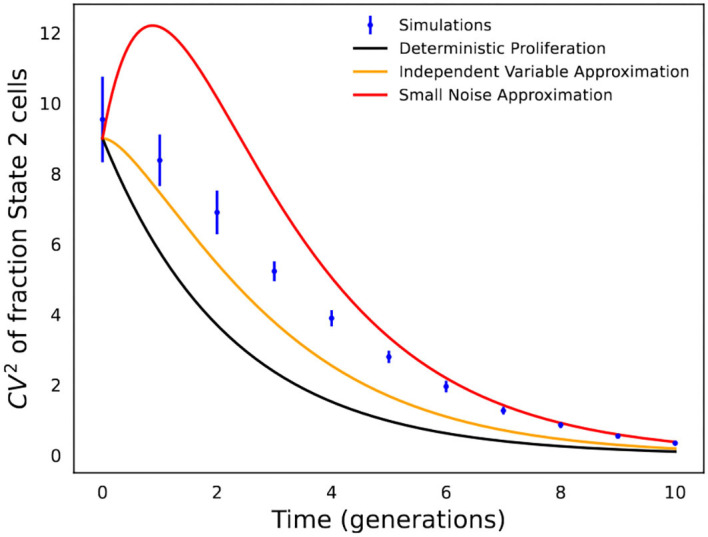
Comparison of analytical formulas predicting fluctuations in state fractions across single-cell colonies. Stochastic simulations of the cell proliferation and switching process were used to perform an *in silico* fluctuation test experiment with *f* = 0.1, *k*_*x*_ = 1, and *k*_2_ = 1/5 (i.e., cells spend an average of 5 generations in State 2). *CV*_*f*_ is computed based on 1,000 colonies, and simulations were repeated 20 times to generate error bars that show one standard deviation. The fluctuation in State 2 fractions across colonies as obtained from stochastic simulations are compared with formulas in Equations (5), (10), and (13).

As seen earlier, for a fixed *f* and *t*, *CV*_*f*_ monotonically increases with slower switching (Figure 3). Moreover, Equation (10) provides a more accurate estimation of the magnitude of inter-colony fluctuations compared with the deterministically formulated Equation (5) (Figure 4).

#### Small noise approximation

Considering small fluctuations in **x**(*t*) and **x**_2_(*t*) around their respective average population counts, one can use the Taylor series to approximate the coefficient of variation of ***f***(*t*) as


(12) $$\begin{array}{l} CV_{f}^{2}\approx \frac{\langle x_{2}^{2}\rangle -{\langle x_{2}\rangle }^{2}}{{\langle x_{2}\rangle }^{2}}+\frac{\langle x^{2}\rangle -{\langle x\rangle }^{2}}{{\langle x\rangle }^{2}}-\frac{2\left(\langle x_{2}x\rangle -\langle x_{2}\rangle \langle x\rangle \right)}{\langle x_{2}\rangle \langle x\rangle }, \end{array}$$

which results in the following formula


(13a) $$\begin{array}{ll} CV_{f}^{2}\left(t\right) & =\left(\frac{2Ze^{\left(-\frac{2k_{x}t}{Z}\right)}-\left(2+Z\right)e^{-k_{x}t}}{Z-2}\right)\frac{1-f}{f}, \end{array}$$

(13b) $$\begin{array}{ll} CV_{f}^{2}\left(t\right) & =\frac{e^{-k_{x}t}\left(1+2k_{x}t\right)\left(1-f\right)}{f},\;Z=2. \end{array}$$

The accuracy of the different formulas in Equations (5), (10), and (13) is shown in Figure 4, which is not surprising, and the formula based on deterministic modeling significantly underestimates fluctuations in State 2 fractions. In contrast, the formulas based on stochastic modeling provide a much better approximation, with Equation (10) (independent variable approximation) performing better at earlier time points when fluctuations are large, while Equation (13) (small noise approximation) works better at longer time points when fluctuations are small.

Recently, Saint-Antoine et al. (2022) performed a benchmarking investigation where *in silico* data were generated using a simulated fluctuation test with 40 single-cell colonies expanded as per a gamma or lognormally distributed cell-cycle time, and matching fluctuations in the data with Equation (10) were used to identify the transient heritability of cell states. This benchmarking indeed shows the utility of this approach in effectively discriminating between fast and slow cell-state switching (Saint-Antoine et al., 2022). However, the estimated average time spent in State 2 was slightly larger than that assumed in the simulations due to the fact that Equation (10) underestimates the actual extent of fluctuations (Figure 4). More rigorous inference approaches are clearly warranted, and a good direction would be to combine formula-based fluctuation-matching with maximum-likelihood approaches that directly fit the model-predicted distribution of state fractions to data.

## 3. Inferring transient heritable across biology systems

Drug resistance in response to targeted therapy is a major obstacle in curing a patient with cancer. The fluctuation test methodology was used to study cancer drug resistance, where single melanoma cells were expanded into colonies for a few weeks and then treated with a chemotherapy drug, vemurafenib (Shaffer et al., 2017). After treatment, each cell was phenotypically classified into two states:

- Drug-sensitive (the cell becomes nonviable in response to treatment).
- Drug-tolerant (the cell survives treatment and later develops into a drug-resistant colony).

Intriguingly, the colony-to-colony fluctuations in the number of surviving cells were significantly larger than a Poisson distribution with Fana factors (variance/mean) reported in the range of ≈10 − 25 (Shaffer et al., 2017). Recalling that the Fana factor of Poisson-distributed random variables is one, we observed that the Fana factors were orders of magnitude smaller than that predicted by a model where drug-tolerant cells arose using an irreversible genetic mutation prior to treatment. These observed fluctuations were consistent with a model of pre-treatment reversible switching between drug-sensitive and drug-tolerant states, and cells in the tolerant state can transform to become drug-resistant after long-term drug exposure (Shaffer et al., 2017; Harmange et al., 2022). In summary, **a rare subpopulation of drug-tolerant melanoma cells (**≈** one out of a thousand cells) are transiently primed to respond to drug therapy even in the absence of the drug**. The study also identified several resistance markers that were expressed in rare cells, and stochastic modeling of interconnected networks of such genes mechanistically captured transient entry and exit from the drug-tolerant state (Schuh et al., 2020). These insights from the fluctuation assay add to the growing understanding of *reversible and non-genetic mechanisms* leading to the survival of drug-tolerant persister cells that are major drivers of therapy relapse across cancer types (Sharma et al., 2010; Raha et al., 2014; Mu et al., 2017; Duy et al., 2021; Rehman et al., 2021).

Combining the colony-to-colony fluctuations in the number of surviving melanoma cells with the formula in Equation (5) revealed a drug-tolerant state with a transient heritability of roughly five to eight generations before melanoma cells switch back to being drug-sensitive (Saint-Antoine and Singh, 2022). From a therapeutic point of view, knowing these rates of switching can aid the design of drug therapy schedules to delay the emergence of cancer drug resistance (Paryad-Zanjani et al., 2021). These mathematical results also facilitated the development of a novel approach, Memory Sequencing (MemorySeq), that identifies all slowly fluctuating expression programs in rare cells (Shaffer et al., 2020). The basic idea of MemorySeq is to perform bulk RNA sequencing on each single-cell lineage and then find all genes that exhibit much higher fluctuations in their expression levels across lineages as compared with its noise control (fluctuations in expression levels across randomly selected cell populations). **MemorySeq facilitated a genome-wide identification of drug-tolerant expression programs** and single-molecule fluorescence *in situ* hybridization (smFISH) confirmed the upregulation of these genes in rare single melanoma cells (Shaffer et al., 2017). **Inferred switching rates were validated in two ways**: i) The drug-tolerant genes identified in MemorySeq were fluorescently tagged at the protein level. Following single cells using time-lapse microscopy indeed showed individual cells stochastically switching to a high-expression state that persisted transiently for several cell divisions (Shaffer et al., 2020); ii) FACS sorted outlier cells with high expression of these genes exhibited enhanced drug tolerance, with both expression and drug-tolerance levels slowly reverting to corresponding levels in the original unsorted population consistent with model-predicted relaxation kinetics (Shaffer et al., 2017).

**Figure 5:**
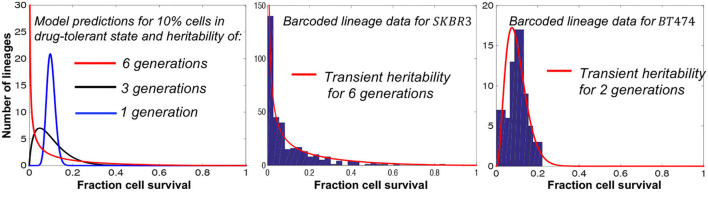
Fluctuation assay on barcoded breast cancer cell lines shows different plasticities for the drug-tolerant state. **Left**: The model predicted fluctuations for different transient heritabilities of the drug-tolerant state. **Right**: The model fits lineage-to-lineage fluctuations in the fraction of cells surviving targeted therapy for two different cell lines (Chang et al., 2021).

Recent advances in barcoding technologies allow single-cell lineage tracing both in culture and *in vivo* (Echeverria et al., 2019; Bowling et al., 2020; Rodriguez-Fraticelli et al., 2020; Leeper et al., 2021; Umkehrer et al., 2021) providing fertile grounds for applications of the fluctuation test. Recently, the fluctuation test has been applied to barcoded data from both melanoma and breast cancer cell lines (Chang et al., 2021; Harmange et al., 2022). In the latter study, barcoded single breast cancer cells were first expanded for several generations and then treated with the tyrosine kinase inhibitor, tucatinib, for 2 weeks. Modeling lineage-to-lineage fluctuations in cell survival revealed a rare subpopulation of drug-tolerant cells that existed even before treatment (Chang et al., 2021). **Interestingly, the transient heritability of the tolerant state significantly differed between cell lines ranging from six generations to only two generations** (Figure 5).

**Figure 6:**
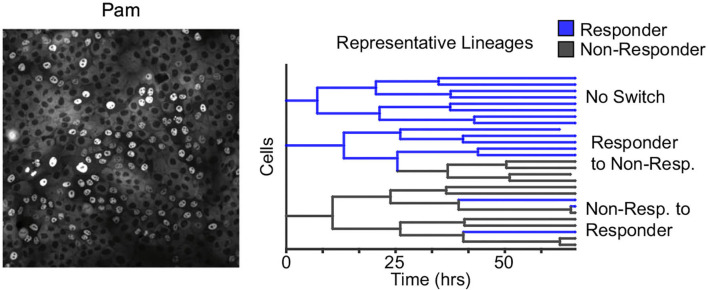
Reversible epigenetic states regulate the innate immune response of single epithelial cells. **Left**: Human epithelial cells when challenged with bacterial microbe-associated molecular patterns (pam) show a digital all-or-nothing activation of NF-κB at the single-cell level. **Right**: The fluctuation test was performed by tracking single-cell derived lineages using time-lapse microscopy and then challenged with pam. The response of single cells was highly correlated within lineages. Subsequent modeling revealed switching between responder and non-responder cell states that in turn are controlled by epigenetic regulation at the promoters of toll-like receptors (Clark et al., 2021).

Along the same theme of reversible non-genetic switching between states, working with a human epithelial cell line time-lapse microscopy to trace single cells within a population to derive lineage trees, these cells were subsequently challenged with microbial signatures (Clark et al., 2021). Data showed a digital all-or-none immune response at the single-cell level, with a subpopulation of cells (≈15%) responding to the challenge (Figure 6). Modeling the number of responders across lineages revealed reversible switching between the responder and non-responder cell states that occurred even before exposure to microbial signatures and was mechanistically mapped to dynamic epigenetic regulation of the toll-like receptor 2 (TLR2) (Clark et al., 2021). Similar digital responses in subpopulations were also seen in primary organoids with individual cells showing switch-like epigenetic modifications of the TLR2 locus (Clark et al., 2021). Cell density can alter the fraction of responding cells, and the fluctuation test was recently used to show that this phenomenon arises from a higher switching rate into responder cell states at lower densities, indicating a form of immune quorum sensing (Antonioli et al., 2018; Van Eyndhoven et al., 2021, 2022).

**Figure 7:**
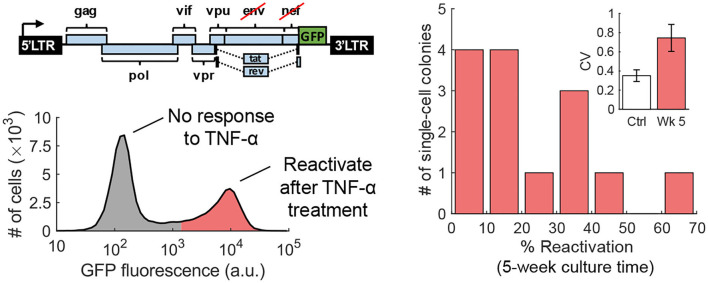
Transient cell state regulates HIV reactivation from latency. **Left**: An isoclonal population of Jurkat cells with a latently infected GFP-tagged HIV-1 gene circuit (one copy of the virus at the same integration site in all cells) when uniformly stimulated with TNF-α causes viral reactivation in a fraction of cells. **Right**: The fluctuation test was performed by first growing single Jurkat cells into colonies and then treating them with TNF-α. The fraction of reactivated cells showed a skewed distribution across colonies consistent with pre-exposure responsive and unresponsive cell states that reversibly switch within several weeks (Lu et al., 2021).

The fluctuation-test approach has also been used to study the reactivation of human immunodeficiency virus (HIV) from latency in T cells (Lu et al., 2021). Latency refers to a dormant state of HIV inside infected cells, and these cells can evade drug treatment, creating a barrier to curing patients (Han et al., 2007; Singh and Weinberger, 2009; Razooky et al., 2015). This study used a specific T-cell line (Jurkat cells) that has a single copy of HIV integrated at the same genome location in all cells. While it is well-known that exposing latent cells to latency reversal agents (LRA) leads to a viral reactivation in a subpopulation (despite cells being isoclonal and receiving the same LRA dosage), it remains a mystery if these cells arise randomly or are a result of a pre-treatment cell state. Performing the fluctuation test at 5 weeks of colony expansion revealed large colony-to-colony fluctuation in the fraction of reactivation cells that was significantly higher than the noise control (Figure 7). However, by 10 weeks of colony growth, the inter-colony fluctuations had attenuated to noise control levels. These data from the fluctuation assay performed for different duration of colony growth were consistent with a model of pre-treatment switching of single cells between the unresponsive and responsive states, with cells residing in the latter state for several weeks (Lu et al., 2021). The long-timescale switching found here indicates an epigenetic mechanism where slow turnover of histone marks at the HIV integration site drives all-or-none reactivation in individual cells. Consistent with this finding, previous studies implicated HIV-promoter methylation patterns as key determinants of viral reactivation in response to LRAs (Blazkova et al., 2009).

## 4. Conclusion

The original Luria–Delbrück fluctuation experiment done 80 years ago revolutionized the field of bacterial genetics and led to an innovative method for estimating mutation rates. In this study, we reviewed a generalization of this approach to elucidate the transient heritability of cellular states, which has important implications for both biology and medicine. The approach relies on using the inter-colony variation, as quantified analytically using *CV*_*f*_ through the various approximations in Equations (5), (10), and (13), together with *a priori* knowledge on the average of fraction cells in different states in Equation (1), to estimate the interconversion rate between two given cellular states. Thus, the kinetics of reversible switching can be identified from the magnitude of fluctuations using a *single endpoint measurement*. In many cases, more information can be acquired by repeating the fluctuation test at different time points of colony expansion (Lu et al., 2021) or the lineage tracking of cells in an expanding colony to know the exact kinship between cells (Veening et al., 2008b; Hormoz et al., 2016; Wheat et al., 2020; Clark et al., 2021; Vertti-Quintero et al., 2022). This can be used to further test the model predictions (for example, the colony-to-colony variation in ***f***(*t*) monotonically decreasing over time) and expand the model by relaxing many of the assumptions made in its formulation. We discussed this latter point in more detail below.

While much of this analytical study used simplifying assumptions, it can be extended along several fronts:

1. **Considering different proliferative potential** of cellular states, which is especially important given that drug persisters can in some cases grow significantly slower than drug-sensitive cells (Balaban et al., 2004; Maisonneuve et al., 2013; Feng et al., 2014; Meouche et al., 2016; Fisher et al., 2017; Manuse et al., 2021).
2. **Inclusion of density-dependent effects** where transition rates themselves depend on the local cell density. For example, the rates *k*_1_ and *k*_2_ can depend on the fraction of cells in State 2 capturing some form of quorum sensing, which is well-known in microbial populations and also reported in immune cells (Antonioli et al., 2018; Van Eyndhoven et al., 2022).
3. **Allowing for irreversible transformations**, as seen in our work with melanoma cells, where reversible drug-tolerant cells proceed irreversibly to a resistant phenotype upon drug exposure (Shaffer et al., 2017), and some recent work has been done in this direction (Bokes and Singh, 2021).
4. **Modeling a continuum of cell states** using a partial differential equation-based framework, with the inference of forward and backward diffusion rates based on the generalized fluctuation test.
5. **More realistic models of colony growth** are needed where the cell-cycle times themselves have multi-generational memory. Preliminary study suggests that this can lead to very different stochastic variations of colony sizes as compared with simplistic approaches where cell-cycle times are considered independent and identically distributed random variables (Nieto et al., 2022).

Relaxing many of these assumptions will result in nonlinear stochastic dynamical systems, which may not be amenable to analytical approaches. In such cases, exact stochastic simulations of the underlying processes can be used to obtain the statistical distributions that can then be fitted to data using a maximum-likelihood approach to infer model parameters.

**An important generalization of this approach will be to infer switching topologies and rates for an arbitrary number of cell states**. There is an increasing body of research that uses expression profiles to classify individual cells in different cell states (Trapnell, 2015; Hormoz et al., 2016; Hejna et al., 2017; Lieberman et al., 2018; Neftel et al., 2019; Andreatta et al., 2021; Li et al., 2021; Shao et al., 2021). While these methods only provide a static distribution of cell states, **fluctuation analysis can shed rich insights into the plasticity of states by characterizing dynamical transitions between them**. To appreciate the complexity of the problem, note that, for *n* cell states, there are 2^*n*(*n*−1)^ possible network topologies. While *n* = 2 yields four topologies (reversible switching, irreversible transitions between either state, and the trivial case of no transitions), this number increases quite sharply to 64 (*n* = 3) and 4,096 topologies (*n* = 4). With multiple cell states, the data obtained from the fluctuation assay are also richer—one measures both the variances and covariances in the fraction of different states across colonies. Given the data from a fluctuation assay, mathematical tools can combine stochastic modeling of state transition with likelihood-based methods to rank all plausible topologies based on their likelihood of occurrence with corresponding transition rates. These predictions can then be used to design further experiments to discriminate between the most probable topologies and to validate the model by sorting cells in a given state and following the redistribution of states over time.

Cognizant of the fact that the fluctuation test requires the expansion of cells, its application in human cells has primarily been in well-established cell line model systems uncovering fundamental questions in cancer biology, immunology, and virology. However, we believe that the true potential of this generalized fluctuation test is in microbial systems, where (unlike mammalian cells) cells are readily grown from single cells with relatively fast doubling times, and there is a longstanding tradition of performing the Luria–Delbrück experiment. Working in this direction, we currently have several ongoing collaborations probing antibiotic tolerance across bacterial species (*Mycobacterium tuberculosis, Vibrio cholerae, Enterobacter cloacae*, and *Salmonella enterica*), and a recent study characterizes the transient priming of bacterial cells even before lethal antibiotic stress (Hossain et al., 2022).

The rapid growth of microbial cells allows for an ingenious experiment to validate the transient heritability of cellular states. More specifically, each single-cell colony is split into two colonies, and one split is assayed for survival to antibiotics, and the other split is assayed after dilution and further growth for several generations. If antibiotic tolerance is transient, then the correlation in bacterial survival between the initial and later splits will weaken as the second split is allowed to grow for more generations before treatment. Moreover, considering several splits of the same population can allow for multiplexing responses to different stress conditions. For example, two splits of the same colony can be treated with two different classes of antibiotics, and the correlation in fraction survival across colonies can point toward similar or different biochemical pathways at work. Finally, one split (both post-treatment and pre-treatment) can also be used for proteomic/transcriptomic/metabolomic studies, which can aid in a mechanistic mapping of stress-tolerant states to expression programs using the MemorySeq approach (Shaffer et al., 2020). In summary, building up on this transformative fluctuation-based tool combined with mechanistic stochastic modeling of underlying gene networks can significantly advance the field of single-cell biology and have tremendous applications across life science disciplines.

## Author contributions

AS and MS-A contributed to the writing and revision of this review article. All authors contributed to the article and approved the submitted version.
